# FANCD2 promotes mitotic rescue from transcription-mediated replication stress in SETX-deficient cancer cells

**DOI:** 10.1038/s42003-022-04360-2

**Published:** 2022-12-21

**Authors:** Maha Said, Viviana Barra, Elisa Balzano, Ibtissam Talhaoui, Franca Pelliccia, Simona Giunta, Valeria Naim

**Affiliations:** 1 CNRS UMR9019, Université Paris-Saclay, Gustave Roussy, 114 rue Edouard Vaillant, 94805 Villejuif, France; 2 Department of Biological, Chemical and Pharmaceutical Sciences and Technologies (STEBICEF), University of Palermo, Viale delle Scienze, 90128 Palermo, Italy; 3 Department of Biology & Biotechnology “Charles Darwin”, University of Rome Sapienza, Piazzale Aldo Moro 5, 00185 Roma, Italy

**Keywords:** Chromosomes, Cancer

## Abstract

Replication stress (RS) is a leading cause of genome instability and cancer development. A substantial source of endogenous RS originates from the encounter between the transcription and replication machineries operating on the same DNA template. This occurs predominantly under specific contexts, such as oncogene activation, metabolic stress, or a deficiency in proteins that specifically act to prevent or resolve those transcription-replication conflicts (TRCs). One such protein is Senataxin (SETX), an RNA:DNA helicase involved in resolution of TRCs and R-loops. Here we identify a synthetic lethal interaction between SETX and proteins of the Fanconi anemia (FA) pathway. Depletion of SETX induces spontaneous under-replication and chromosome fragility due to active transcription and R-loops that persist in mitosis. These fragile loci are targeted by the Fanconi anemia protein, FANCD2, to facilitate the resolution of under-replicated DNA, thus preventing chromosome mis-segregation and allowing cells to proliferate. Mechanistically, we show that FANCD2 promotes mitotic DNA synthesis that is dependent on XPF and MUS81 endonucleases. Importantly, co-depleting FANCD2 together with SETX impairs cancer cell proliferation, without significantly affecting non-cancerous cells. Therefore, we uncovered a synthetic lethality between SETX and FA proteins for tolerance of transcription-mediated RS that may be exploited for cancer therapy.

## Abstract (web summary)

A synthetic lethal interaction is reported between the R-loop helicase Senataxin (SETX) and Fanconi anemia pathway proteins, with SETX deficiency increasing replication stress-induced chromosome fragility & activation of mitotic DNA synthesis.

## Introduction

DNA replication is an essential process that must be tightly regulated and completed ahead of chromosome segregation to maintain genome stability and sustain cell proliferation.

However, the cell is often faced with circumstances that challenge genome duplication due to stalling of replication forks and alterations in replication dynamics, a condition known as ‘replication stress’ (RS)^1^. RS can be the consequence of DNA damage induced by exogenous or endogenous genotoxic stress. Although the mechanisms that protect cells against exogenous sources of RS have been extensively characterized, how endogenous RS is dealt with by the cell is still elusive. Even in the absence of external assaults, replication forks can be hindered by lesions generated as a consequence of cellular metabolism and non-canonical DNA structures such as G-quadruplexes, hairpins, RNA:DNA hybrids or R-loops (three-stranded structures composed of an RNA − DNA hybrid and a displaced single-stranded DNA) that may be formed as a result of TRCs^2,3^. To allow faithful genome replication in the face of RS and avoid mitotic division with under-replicated DNA, cells possess protective mechanisms that stabilize, remodel or restart stalled replication forks, while delaying cell cycle progression^4^. However, a large body of evidence has recently shown that under low levels of RS, cells may not resume stalled replication forks and complete genome replication until late G2 or even mitosis^5–8^. In particular, delayed replication completion often occurs at genomic loci, such as fragile sites, telomeres and centromeres, that are intrinsically difficult-to-replicate^9–12^. At these loci, a number of pathways have been shown to process and resolve different kinds of replication or repair intermediates that result in sister chromatid interlinkage, thus promoting mitotic rescue from replication stress and proper chromosome segregation^9,13,14^. The FA pathway, deficient in the chromosomal instability disorder Fanconi anemia, is one such pathway that maintains genome stability in response to RS, by acting throughout S-phase and mitosis to promote complete genome replication and faithful chromosome segregation during anaphase^15^.

The FA proteins play key roles in the repair of DNA interstrand cross-links, protection of stalled replication forks and in coordination of replication and transcription^16–19^. FA cells exhibit a high rate of chromosomal aberrations (i.e., breaks, gaps, and radial figures), due to compromised or aberrant repair of replication-coupled DSBs. Aberration breakpoints in FA patients frequently occur at common fragile sites (CFS)^20^, late replicating genomic regions that host large genes^21,22^ and tend to form gaps and breaks on mitotic chromosomes upon induction of RS, notably after treatment with aphidicolin (APH), an inhibitor of DNA polymerase alpha^23^. FANCD2, the key activated target of the FA pathway, together with other repair factors such as BLM and the components of structure specific endonucleases (SSE) SLX1-SLX4, XPF-ERCC1 and MUS81–EME1 promotes the resolution of replication intermediates that persist at CFS in mitosis^24–26^. Under-replicated DNA structures processed by SSE are detected as gaps or breaks on mitotic chromosomes and undergo DNA synthesis mediated by PolD3 to allow complete replication in a process named mitotic DNA synthesis (MiDAS)^7^. Moreover, if these structures persist or are mis-repaired, they can lead to chromosome segregation defects and cell death^25^. FANCD2 specifically localizes to CFS loci^27,28^ that remain incompletely replicated in mitosis^24,29^, in a manner dependent on transcription and mitochondrial stress^30^, where it facilitates their replication and limits the formation of R-loops through a yet unidentified mechanism^31^, likely by interacting with RNA processing factors^32^. Recently, it has been reported that interaction between the FA protein SLX4/FANCP and the DNA helicase RTEL1 promotes FANCD2 foci formation at sites of transcription-replication encounters to prevent transcription-mediated RS^33^. These works highlight that transcription and R-loops represent a significant source of RS that requires FANC proteins to act on, but how the FA pathway resolves this type of endogenous replication blockage and the outcome of unresolved transcription-associated RS is unknown.

R-loops tend to occur naturally over specific loci, such as at gene promoters and terminator regions, where they play key regulatory roles^34^. However, R-loops pose a threat to the genome when they occur in an unscheduled manner, so that their prevalence must be continuously monitored by specific factors that contribute to their resolution. R-loops can be dismantled through several activities including the endoribonuclease RNase H, the translocase FANCM, and helicases such as Aquarius and Senataxin^35^.

Senataxin (SETX) is an RNA:DNA helicase mutated in Axonal neuropathy type 2 (AOA2) and Amyotrophic Lateral Sclerosis type 4 (ALS4) patients^36,37^, with functions at the interface between transcription and genome maintenance pathways^35,38–43^. Recent work has demonstrated that SETX and its yeast ortholog Sen1 play a key role in resolution of R-loops arising from TRCs, promoting replisome progression through transcribed genes^43–46^. However, the relevance of SETX function in cancer cells that sustain intrinsic RS^47^ has not been fully established.

Here, we show that FANCD2 is necessary to counteract endogenous RS and allow proliferation of SETX-deficient cancer cells. Our results demonstrate that the absence of SETX increases spontaneous chromosome fragility due to transcription-associated RS that persists in mitosis. Furthermore, we show that SETX-depleted cells activate MiDAS in a FANCD2-, XPF- and MUS81-dependent manner to rescue under-replicated DNA. Importantly, depletion of both FANCD2 and SETX greatly reduces cell survival as a result of accumulated segregation defects. Collectively, our data demonstrate that continuous cycling of SETX-deficient cells relies on FANCD2-mediated rescue of endogenous RS and chromosome under-replication during mitosis, underscoring the possibility to exploit a novel synthetic proliferation defect for cancer therapy.

## Results

### SETX prevents spontaneous chromosome fragility marked by recruitment of FANCD2 in mitosis

**Fig. 1:**
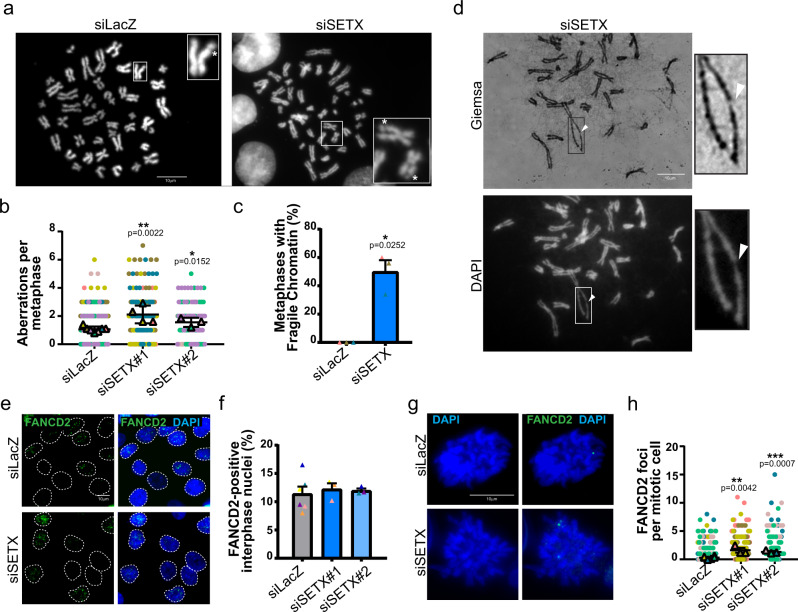
SETX depletion triggers chromosome fragility and recruitment of FANCD2 in mitotic cells. **a** Representative images of metaphases from control (LacZ) and SETX siRNA-transfected cells. Chromosomes exhibiting gaps are magnified in insets. **b** Quantification of chromosome aberrations (gaps or under-condensed regions) on mitotic chromosomes from control, siSETX#1 and siSETX#2 siRNA-transfected cells. **c** Quantification of metaphases showing fragile chromatin in control and SETX siRNA-transfected cells. **d** Representative metaphase from the SETX-deficient condition stained with Giemsa (upper panel) and DAPI (lower panel) showing the fragile chromatin phenotype characterized by unfolded chromatin at the level of weakly DAPI-stained chromatid arm. **e** Representative example of immunofluorescence analysis of FANCD2 foci in interphase nuclei from control or SETX-depleted cells. **f** Quantification of the percentage of FANCD2 positive (>5 FANCD2 foci) interphase cells. **g** Representative example of immunofluorescence analysis of FANCD2 foci in mitotic cells from control or SETX-depleted condition. **h** Quantification of the number of FANCD2 foci in mitotic cells.

Knowing that SETX is required to avoid TRCs^43,44^ and R-loop accumulation^38,45,48,49^, we asked whether SETX depletion in otherwise unchallenged conditions may induce transcription-mediated RS, and its consequences for genome stability^33^. For that, we carried out cytogenetic analysis of metaphase spreads prepared from SETX-depleted and control conditions. We detected a significant increase in spontaneous chromosome fragility in SETX-depleted cells as compared to control HCT116 colon cancer cells (Fig. 1a, b). We confirmed this result using two different siRNAs targeting SETX (Supplementary Fig. 1a). Strikingly, SETX depletion induced a peculiar type of chromosome aberrations that we defined as “fragile chromatin”, characterized by chromatid gaps or under-condensed regions that differ from canonical chromosome gaps/breaks because it is often associated with chromatin loops extruding from the main chromosome axis, visible on Giemsa-stained chromosomes (Fig.1c, d). To confirm that SETX depletion was associated with increased chromosome fragility, we performed cytogenetic analysis in both HeLa and RPE-1 cells. Similar to HCT116 cells, chromosomes from HeLa cells exhibited more aberrations in the absence of SETX compared to the control counterparts (Supplementary Fig. 1b). Remarkably, the absence of SETX did not induce chromosome fragility in the non-cancerous RPE-1 cells (Supplementary Fig. 1c). This different behavior was not due to decreased knockdown efficiency (Supplementary Fig. 1d, e). The fragile chromatin phenotype we identified resembles classical chromatid gaps, discontinuities in DNA structure detected as DAPI-negative regions in metaphase chromosomes, possibly underlying under-replicated or unresolved DNA structures. Therefore, we looked at the presence of FANCD2, that is recruited to genomic loci that remain under-replicated in mitosis^24^. We performed immunofluorescence analysis to reveal FANCD2 foci assembly in asynchronous growing cells after siRNA-mediated SETX-knockdown. FANCD2 monoubiquitylation and protein levels were unchanged in the absence of SETX (Supplementary Fig. 1a). The cell cycle progression was not significantly affected in SETX-depleted cells (Supplementary Fig. 1f). Moreover, we did not detect significant differences between control and SETX-knockdown cells in the fraction of FANCD2-positive nuclei in interphase (Fig. 1e, f). However, when we looked at mitotic cells, we detected a significant increase in spontaneous FANCD2 foci on mitotic chromosomes in SETX-depleted cells (Fig. 1g, h).

Depletion of SETX also induced a significant increase in the number of FANCD2 mitotic foci in HeLa cells (Supplementary Fig. 1g). In contrast, and in line with the absence of spontaneous chromosome fragility, SETX depletion did not induce FANCD2 mitotic foci formation in RPE-1 cells (Supplementary Fig. 1h). This may be due to a higher level of endogenous RS in cancer cells^47^.

**Fig. 2:**
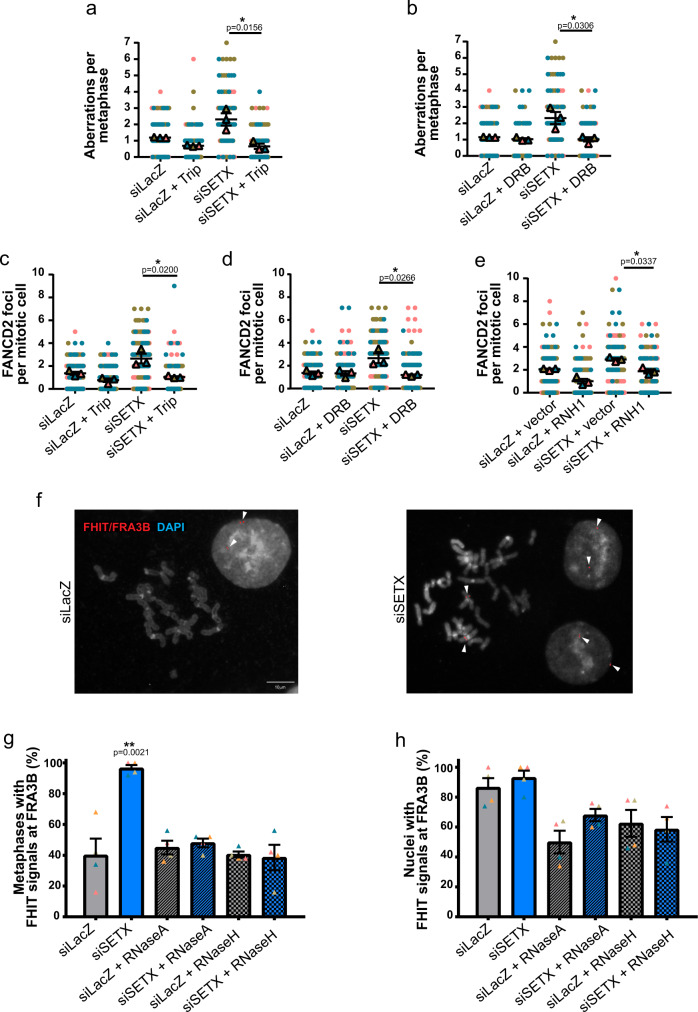
FANCD2 foci in mitotic cells following SETX depletion are dependent on transcription and persistent R-loops. Quantification of the number of chromosome aberrations per metaphase after treatment with 1 µM Triptolide (Trip) for 8 h (**a**), or 20 µM DRB for 16 h (**b**) in control (LacZ) and SETX-depleted cells. Quantification of the number of FANCD2 foci in mitotic cells after treatment with 1 µM Triptolide (Trip) for 8 h (**c**), or 20 µM DRB for 16 h (**d**), or following transfection of an empty vector or a plasmid over-expressing RNaseH1 (RNH1) in control and SETX-depleted cells (**e**). **f** Examples of signals detected after combined RNA-DNA FISH with probes for FHIT/FRA3B in control and SETX-depleted cells. Representative metaphases and interphase nuclei are shown. Quantification of FHIT/FRA3B signals in metaphase (**g**) and interphase (**h**) in control or SETX-depleted cells. Signals recorded on slides prior or after treatment with RNase A and RNase H1 are indicated.

To understand the origin of mitotic FANCD2 foci observed in the absence of SETX, we asked whether they may be due to transcription-mediated RS. To this end, we used 2 transcription inhibitors, Triptolide (Trip) and 5,6-dichloro-1-beta-D-ribofuranosylbenzimidazole (DRB) in control and SETX-depleted cells, to inhibit transcription initiation or the release of paused RNA pol II into active elongation, respectively. Transient inhibition of transcription did not affect FANCD2 protein levels (Supplementary Fig. 2a). Remarkably, both treatments fully rescued chromosome aberrations and the number of FANCD2 foci in mitosis in SETX-depleted cells to control levels (Fig. 2a–d). Since SETX plays a key role in removing R-loops during replication^45^ and FANCD2, amongst other FANC proteins, is involved in limiting R-loops emerging from transcription-replication conflicts^16,19,31^, we hypothesized that FANCD2 foci formed in the absence of SETX were dependent on R-loops. To address this, we transfected control and SETX-depleted cells with an empty vector or a plasmid over-expressing RNase H1 (RNH1) (Supplementary Fig. 2b). RNase H1 over-expression decreased the number of FANCD2 foci in both control and SETX-depleted mitotic cells (Fig. 2e), confirming its recruitment to these structures^50^. This also suggests that endogenous stress in SETX-depleted cancer cells likely reflects transcription-associated RS resulting in R-loops. To further understand the nature of the FANCD2 foci emerging in SETX-depleted mitotic cells, we wanted to determine whether their assembly was sensitive to low doses of RS induced by APH. Strikingly, we found no significant difference in the percentage of APH-induced FANCD2 foci in the presence or absence of SETX, indicating that the structures induced by SETX depletion are distinct from those resulting from APH-induced RS (Supplementary Fig. 2c). Taking these results together, our data suggests that appearance of under-condensed, fragile chromatin regions, due to SETX depletion, likely correspond to persistent R-loops resulting from unresolved TRCs as opposed to late replication intermediates resulting from APH-induced fork slowing.

Since FANCD2 targets CFS loci even in unperturbed conditions^24,30,51^, we directly assessed for the presence of CFS-associated nascent transcripts that would reveal persistent R-loops manifesting in the cytological phenotype observed in SETX-deficient cells. We performed simultaneous RNA-DNA fluorescence-in-situ-hybridization (FISH) to detect FHIT transcripts associated to FRA3B, the most fragile CFS in HCT116 cells^21^. Interestingly, we detected increased FHIT foci associated to the FRA3B locus in metaphase chromosomes of SETX-deficient cells (Fig. 2f, g). These foci were reduced to control levels after treatment with both RNAse A and RNAse H, indicating that signals correspond to RNA molecules. Similar results were obtained when FISH signals were scored on slides pre-treated with an RNAse H containing solution or when cells were transfected with an RNAse H1 over-expressing plasmid (Supplementary Fig. 2d). We did not detect significant differences in the percentage of interphase nuclei with FRA3B-associated FHIT RNA foci between SETX-depleted and control cells (Fig. 2f, h). These data indicate that SETX is necessary to prevent transcription-dependent R-loops within the FRA3B locus that are targeted by FANCD2 during mitosis.

### FANCD2 promotes rescue of under-replicated DNA and prevents chromosome mis-segregation in SETX-depleted cells

**Fig. 3:**
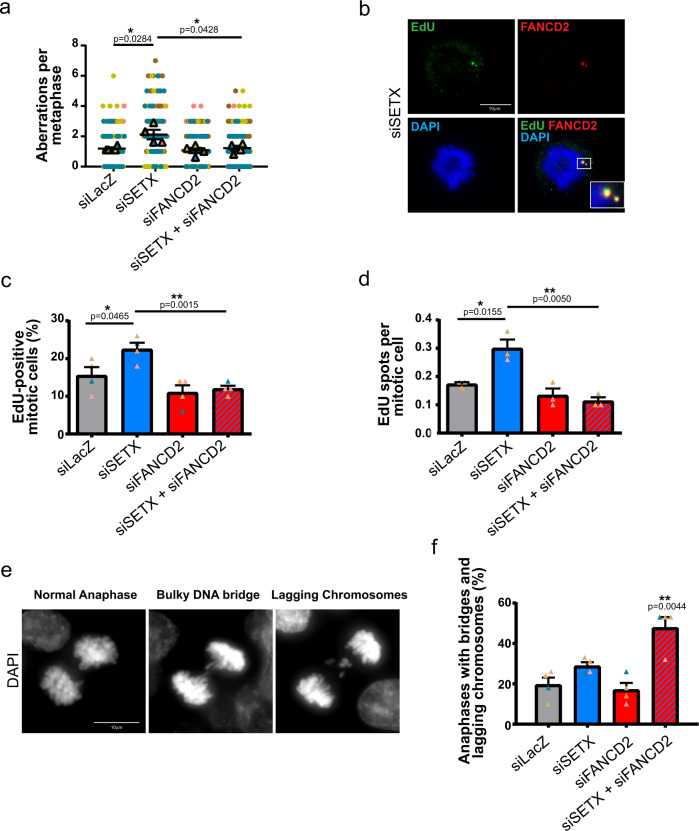
FANCD2 promotes MiDAS and prevents chromosome segregation defects in SETX-depleted cells. **a** Quantification of chromosome aberrations in control (LacZ), SETX, FANCD2 and SETX and FANCD2 siRNA-transfected cells. **b** Representative metaphase from SETX-depleted condition labeled with EdU (green) and stained for FANCD2 (red) showing EdU incorporation in mitotic chromosomes colocalizing with FANCD2. DNA is counterstained with DAPI (blue). **c** Quantification of the percentage of metaphases showing EdU incorporation in control (LacZ), SETX, FANCD2, or SETX and FANCD2 siRNA-transfected cells. **d** Quantification of the number of EdU signals (spots) in mitotic cells from control, SETX, FANCD2, or SETX and FANCD2 siRNA-transfected condition. **e** Representative examples from immunofluorescence analysis of chromosome segregation at anaphase showing normal chromosome segregation, anaphase bridges, and lagging chromosomes. DNA was counterstained by DAPI to show bulky DNA bridges and lagging chromatin. **f** Quantification of bulky DNA bridges and lagging chromosomes from control, SETX, FANCD2, and SETX and FANCD2 siRNA-transfected cells.

Under-replicated DNA, resulting from RS, that bypasses the checkpoint and persists up to mitosis may lead to sister-chromatid nondisjunction and chromosome segregation defects^25,29,52^. Notably at CFSs, the mitotic processing and resolution of late replication intermediates that promotes the cytogenetic appearance of gaps and breaks (resulting in “CFS expression”) is a last attempt to limit genome instability ahead of chromosome segregation^13^. To understand whether FANCD2 has a role in resolving under-replicated DNA in SETX-deficient cells, we co-depleted FANCD2 with SETX (Supplementary Fig. 3a) and looked at the frequency of chromosome aberrations. Interestingly, while the chromatid gaps observed after SETX-depletion were fully suppressed by co-depleting SETX and FANCD2 (Fig. 3a), fragile chromatin was increased by loss of each protein and more so by depletion of both proteins (Supplementary Fig. 3b). These results suggest that, while suppressing fragile chromatin, FANCD2 also promotes the processing of under-replicated DNA generated by the loss of SETX function.

RS delays replication completion and activates MiDAS through a mechanism akin to break-induced replication (BIR) at difficult-to-replicate loci including fragile sites and telomeres^7,53,54^. To determine if chromosome gaps observed after SETX depletion results from regions undergoing MiDAS, we performed EdU incorporation followed by Click-iT reaction and scored cells with 1 or more EdU focus/foci as “EdU positive” since EdU incorporation in mitosis is a rare event under untreated conditions (Fig. 3b)^55^. Upon evaluation of the percentage of mitotic cells that have incorporated EdU in SETX, FANCD2, and SETX and FANCD2 co-depleted cells, we found a significant increase in EdU positive mitotic cells after SETX-depletion compared to control cells (Fig. 3c). FANCD2 down-regulation had no significant effect on MiDAS compared to the control condition. However, in the absence of FANCD2 the increased EdU incorporation in SETX-deficient cells was completely suppressed (Fig. 3c). We also assessed the number of EdU spots on mitotic chromosomes and found a significant increase in SETX-depleted mitotic cells, which was again suppressed by co-depletion with FANCD2 (Fig. 3d). Hence, SETX-depletion actively induces MiDAS that is dependent on FANCD2. Consistently, we observed that FANCD2 foci colocalized with EdU on mitotic chromosomes (Fig. 3b).

To confirm the functional role of FANCD2 in rescuing under-replicated DNA structures generated as a consequence of SETX-deficiency, we examined the percentage of anaphase cells harboring DAPI-stained DNA bridges and lagging chromosomes (Fig. 3e). FANCD2 or SETX siRNA mediated depletion did not significantly affect the frequency of cells with anaphase bridges and lagging chromosomes. Interestingly, co-depleting SETX and FANCD2 led to a synergistic increase in the percentage of cells presenting chromosome segregation defects when compared to control and to the single-depleted cells (Fig. 3f). These results indicate that FANCD2 limits chromosome segregation defects in SETX-deficient cells. In addition, our data implies that chromosome fragility observed in SETX-depleted cells results from active processing of unresolved DNA structures in mitosis that is promoted through the recruitment of FANCD2.

### MUS81 and XPF promote processing of unresolved DNA structures and MiDAS in SETX-depleted cells

**Fig. 4:**
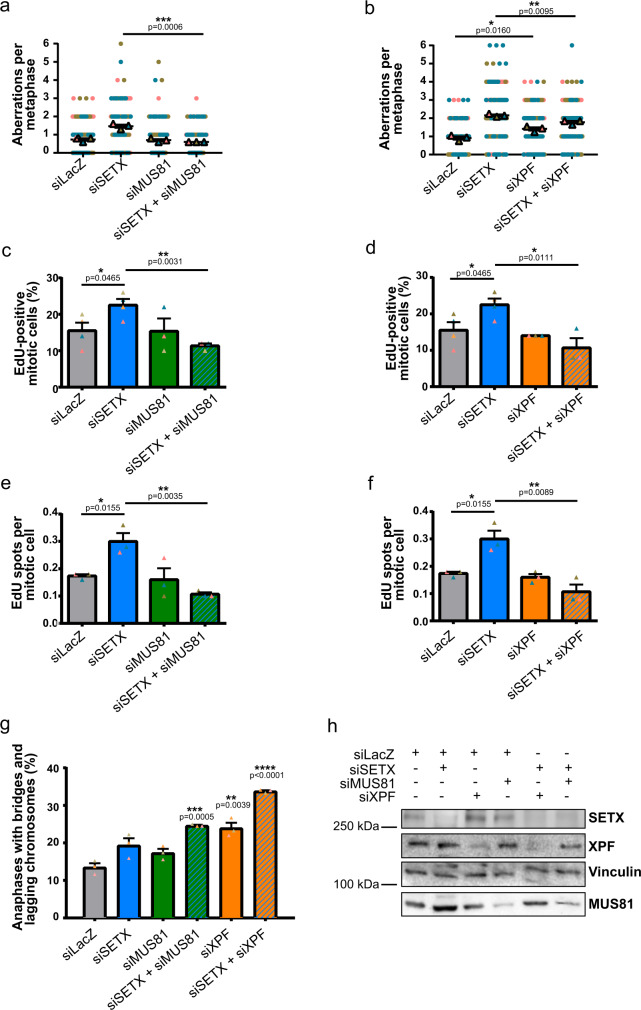
Chromosome aberrations and MiDAS in SETX-depleted cells depend on XPF and MUS81. **a** Quantification of gaps or breaks on mitotic chromosomes from control (LacZ), SETX, MUS81, and SETX and MUS81 siRNA-transfected cells. Gaps and breaks are defined as discontinuities in DNA staining of a size that is lesser or greater with respect to the thickness of the chromatid arm, respectively. **b** Quantification of gaps or breaks on mitotic chromosomes from control, SETX, XPF, and SETX and XPF siRNA-transfected cells. Gaps and breaks are defined as described above. **c** Quantification of the percentage of metaphases showing EdU incorporation in control, SETX, MUS81, and SETX and MUS81 siRNA-transfected cells. **d** Quantification of the percentage of metaphases showing EdU incorporation in control, SETX, XPF, and SETX and XPF siRNA-transfected cells. **e** Quantification of the number of EdU spots in mitotic cells from control, SETX, MUS81, and SETX + MUS81 siRNA-transfected condition. **f** Quantification of the number of EdU spots in mitotic cells from control, SETX, XPF, and SETX + XPF siRNA-transfected condition. **g** Frequency of anaphases presenting DNA bridges and lagging chromosomes in control, SETX, MUS81, SETX + MUS81, XPF, and SETX + XPF siRNA-transfected cells. **h** Western blot showing depletion of the corresponding proteins after siRNA-mediated knockdown of SETX, XPF, MUS81, SETX and XPF, and SETX and MUS81.

Replication forks stalled by transcription complexes with subsequent R-loops accumulation are considered a threat to the genome in that they can be targeted by SSE^56–59^, yet the controlled activity of SSE during mitosis is required to ensure faithful completion of replication^58,60^. We then wanted to assess whether the chromosome aberrations observed in SETX-deficient cells were dependent on either of the two SSE, MUS81 or XPF, that process stalled replication forks and R-loops, respectively^56,57,61,62^. We performed metaphase spreads and scored the frequency of chromosome gaps or breaks in control, SETX-, MUS81-, XPF-, and SETX and MUS81 or SETX and XPF co-depleted cells (Supplementary Fig. 4a, b). RNAi-mediated depletion of MUS81 did not affect chromosome aberrations as compared to control condition (Fig. 4a). Interestingly, co-depletion of MUS81 in SETX-depleted cell completely suppressed the spontaneous chromosome fragility induced by SETX deficiency (Fig. 4a). On the other hand, we found that XPF depletion increased the number of breaks and gaps compared to control (Fig. 4b), though to a lesser extent than in SETX-depleted condition. However, co-depleting XPF with SETX significantly decreased the frequency of aberrations observed in SETX-depleted cells (Fig. 4b). Taken together, these data indicate that MUS81 is implicated in the induction of chromosome gaps detected in SETX-depleted cells. XPF, on the other hand, participates in the formation of a significant fraction of the chromosome gaps caused by the absence of SETX while also limiting chromosome fragility per se.

Next, we measured EdU incorporation in mitotic cells to determine if SSE-mediated processing promotes MiDAS in SETX-deficient cells. We detected no significant change in the percentage of EdU positive mitotic cells in MUS81-depleted cells under untreated conditions (Fig. 4c), in agreement with what has been previously reported^63^. However, depletion of MUS81 in SETX-deficient cells decreased the percentage of EdU positive mitotic cells to levels similar to the control (Fig. 4c). Similar to MUS81, co-depleting XPF with SETX also significantly reduced the percentage of mitotic cells showing EdU incorporation with respect to SETX-depleted cells (Fig. 4d). From these results, we conclude that EdU incorporation in mitotic cells is dependent on both MUS81 and XPF endonuclease activities. We further assessed the number of EdU spots on mitotic chromosomes. EdU incorporation in mitotic chromosomes of SETX-deficient cells was completely suppressed by concomitant down-regulation of either MUS81 or XPF (Fig. 4e, f). These data indicate that both MUS81 and XPF are required to process structures arising from unresolved TRCs and promote of MiDAS in SETX-deficient cells. Co-depleting XPF or MUS81 with SETX also increased the frequency of anaphase cells presenting DNA bridges or lagging chromosomes (Fig. 4g, h). Therefore, endogenous RS elicited by SETX deficiency is rescued in mitosis through recruitment of FANCD2 that promotes its resolution through the coordinated activities of XPF and MUS81 endonucleases to ensure faithful chromosome segregation.

### Co-depletion of FANCD2 and SETX synergistically reduces cancer cell proliferation

**Fig. 5:**
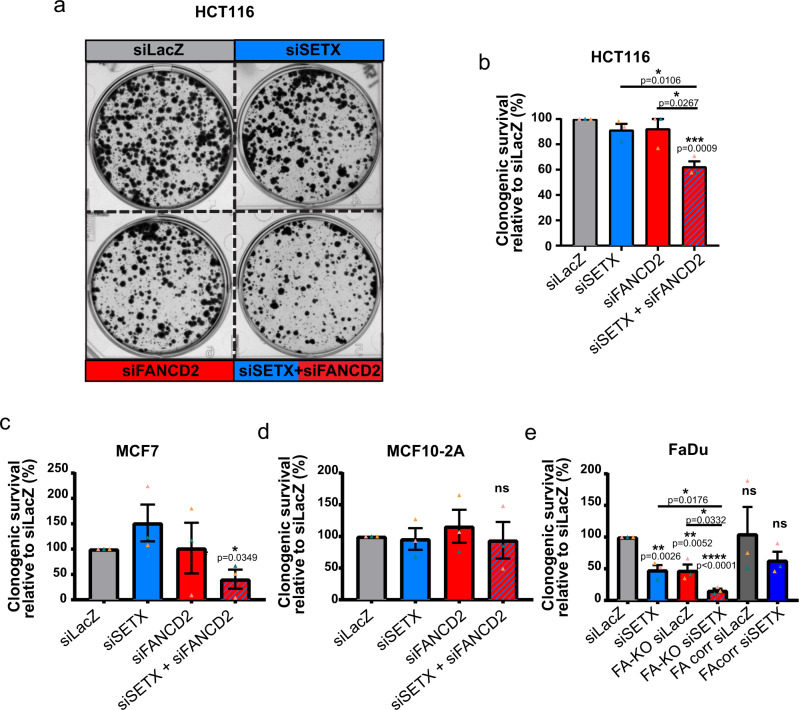
FANCD2 and SETX co-depletion causes a synthetic proliferation defect in cancer cell lines. **a** Representative images of 6-well plates seeded with control (LacZ), SETX, FANCD2, and SETX and FANCD2 siRNA-transfected HCT116 cells and analyzed for clonogenic survival assays. Colonies were stained after 12 days with crystal violet. **b** Quantification of the colonies counted in SETX, FANCD2, and SETX and FANCD2 knockdown HCT116 cells relative to control. **c** Quantification of the colonies counted in SETX, FANCD2, and SETX and FANCD2 knockdown MCF7 breast cancer cells relative to control. **d** Quantification of the colonies counted in SETX, FANCD2, and SETX and FANCD2 knockdown MCF10-2A mammary epithelial cells relative to control. **e** Quantification of the colonies counted in LacZ or SETX knockdown FaDu, FaDu-KO (FANCA^−/−^) or FA-corr (FA-KO + FANCA) head and neck carcinoma cells relative to control (siLacZ) FaDu cells.

The above results show that SETX and FANCD2 cooperate under unchallenged conditions to prevent transcription-mediated RS from compromising completion of genome replication and chromosome segregation during mitosis. To determine the impact of this relationship on cell proliferation, we analyzed the clonogenic potential of SETX-, FANCD2-, or SETX- and FANCD2-depleted cells. In HCT116 cells, SETX or FANCD2 depletion did not lead to significant decrease in the clonogenic capacity of cells. However, SETX and FANCD2 co-depletion synergistically reduced the colony forming ability compared to both the control and single depleted counterparts (Fig. 5a, b). This result confirms the relevance of the observed functional cooperation to RS tolerance and mitotic cell division, and highlight a synthetic loss-of-fitness interaction between FANCD2 and SETX. Interestingly, this relationship appears pertinent to cancerous cell lines, like HCT116 and HeLa, that exhibit a similar phenotype of increased endogenous replicative stress associated with FANCD2 foci and chromosome fragility, whereas the non-cancerous RPE-1 cells are not affected by SETX-depletion. Because our data point to a synthetic lethality that may be specific to cancer cells and therapeutically exploited, we set out to extend our analyses to other cell lines and members of the FA pathway. We compared the clonogenic survival of non-tumorigenic breast epithelial (MCF10-2A) and breast cancer (MCF7) cell lines after single or double depletion of SETX and/or FANCD2, and that of parental *FANCA*-proficient, *FANCA* knock-out (FA-KO) or *FANCA* corrected (FA-corr) head and neck cancer (FaDu) cells after depletion of SETX. As shown in Fig. 5c, d double depletion of FANCD2 and SETX only impaired the proliferation of MCF7 cells, sparing non-tumorigenic MCF10-2A. Indeed, double depletion of FANCD2 and SETX in MCF7 cells induced high levels of mitotic abnormalities (Supplementary Fig. 5). Furthermore, SETX depletion almost completely blocked the proliferation of FA-KO FaDu cells, as compared to parental cells, while tumor cell growth was fully rescued in FA-corr cells (Fig. 5e). Taken together, these results show that SETX and FA proteins act upon similar structures that form as a consequence of TRCs where they can back each other up to sustain endogenous transcription-associated RS in cancer cells.

## Discussion

DNA replication and transcription are two vital cellular processes that, when occurring concomitantly, must be tightly regulated to prevent transcription-mediated RS and DNA damage. Indeed, even without DNA damage due to exogenous sources, RS can result from conflicts between replication and transcription machineries and from the generation of R-loops.

Despite increasing evidence that transcription is a primary source of replication impairment and genome instability^2^, much remains to be understood about the structures that are formed when these two processes interfere with each other, whether R-loops are the cause or consequence of RS and DNA damage^3^, and how the multiple factors and pathways that prevent or resolve them gain access to specific structures or genomic loci during the cell cycle^64^. Addressing these issues is crucial to harnessing RS and identifying cancer cell vulnerabilities.

For instance, Sen1/SETX interacts with the replication machinery and removes harmful RNA:DNA hybrids in S-phase^44,45^, while FANCD2 is known to bind to R-loops^50^, and to prevent transcription-mediated RS in a SLX4-dependent manner^33^. Hence, we wanted to determine whether FANCD2 and SETX may act in similar or partly redundant pathways that functionally cooperate at the interface of transcription and replication.

In our study, we show that SETX depletion induces fragile chromatin on mitotic chromosomes, a specific phenotype seen as chromosome aberrations associated with an altered chromatin structure, which differs from the APH-induced gaps and breaks. Accordingly, SETX depletion induces chromosome fragility independent of exogenous RS induced by APH. Similar to APH-induced CFS expression, the phenotypic expression of chromosome fragility is dependent on persistence of unresolved DNA structures and under-replicated DNA. However, in contrast to APH, the phenotype observed in the absence of SETX is dependent on active transcription and on R-loops that persist on mitotic chromosomes (Fig. 2f). Our findings are consistent with recent works suggesting that R-loops are a source of endogenous RS contributing to CFS fragility in unchallenged conditions but are not a major determinant of APH-induced chromosome fragility^65,66^.

Furthermore, we provide evidence that under-replicated DNA resulting from persistent transcription-dependent R-loops requires a distinct pathway for its resolution.

When cells enter mitosis with under-replicated DNA regions, stalled replication forks or late replication intermediates can be cleaved, resulting in cytologically visible gaps or breaks on mitotic chromosomes that are later repaired allowing cells to complete replication and to divide. On the other hand, if under-replicated DNA is not properly processed, it may give rise to joint molecule intermediates and lead to sister-chromatid non-disjunction that cause chromosome mis-segregation and eventually cell death^25,67^. Here we show that FANCD2 recruitment to mitotic chromosomes, in promoting the appearance of gaps in SETX-depleted cells allows to rescue under-replicated DNA and circumvent toxic structures that cause mitotic failure. Accordingly, we detected a synergistic increase in segregation defects in SETX and FANCD2 co-depleted cells.

Recently, studies have shown that the under-replicated DNA that is cleaved in mitosis is then repaired by a BIR-like mechanism that is initiated by SSE-mediated cleavage of under-replicated DNA and DNA synthesis in a POLD3-dependent manner, in MiDAS^7,54^. In line with this, we detected FANCD2-dependent increase in mitotic EdU incorporation in SETX-depleted cells. Interestingly, we also show that SETX is dispensable for MiDAS. MiDAS was shown to depend mainly on the SSE MUS81-EME1 and SLX1-SLX4 that are required to cleave the under-replicated DNA as a precursor for resumption of DNA synthesis^25,54,67,68^. In agreement with this, by depleting MUS81, we reduced the increased DNA gaps in SETX-depleted cells, while concurrently reducing EdU incorporation in mitotic cells.

Furthermore, ERCC1 the regulatory partner of XPF is also implicated in promoting the cleavage of APH-induced replication intermediates, that is required to allow sister-chromatid disjunction^25^. However, we showed that the activity of XPF was not required to resolve structures formed as consequence of APH-induced RS, suggesting that ERCC1 may have a structural or regulatory role on XPF function, and indirectly on the activity of SLX4 and MUS81^25,26^. In our conditions, XPF depletion significantly induced chromosome gaps as compared to control cells, while co-depleting XPF with SETX significantly reduced the SETX-elicited fragility, yet it did not bring it down to control levels. Since XPF plays an important role in R-loop processing, more so in the absence of helicases such as SETX and Aquarius^57^, we hypothesize that the breaks seen in XPF single depletion are likely not BIR-like intermediates since we did not detect increased mitotic EdU incorporation. On the other hand, co-depletion of SETX and XPF reduces the formation of chromosome aberrations as compared to SETX, implying that a subset of gaps detected in SETX depleted cells is dependent on XPF. Consistent with this, XPF is required to promote EdU incorporation in SETX-depleted cells suggesting that the structures that persist in mitosis following loss of SETX function need XPF processing to promote MiDAS. Hence, our study shows for the first time that XPF is required for MiDAS under this specific condition.

**Fig. 6:**
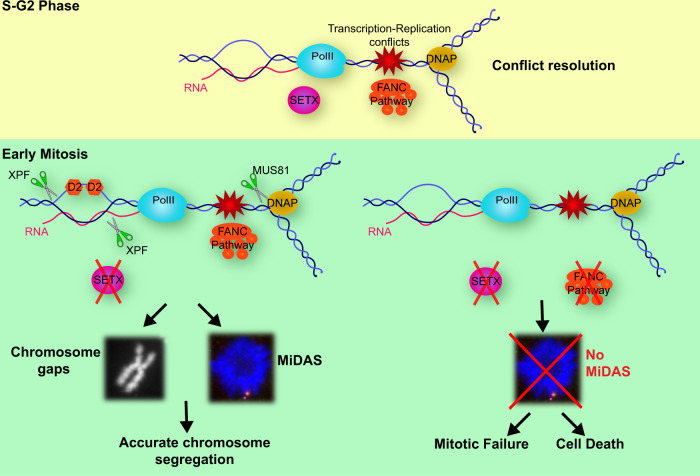
Model for FANCD2-mediated mitotic rescue of transcription-associated RS in SETX deficient cells. SETX and proteins in the FANC pathway constitute partially redundant pathways to resolve transcription-replication conflicts and R-loops. In absence of SETX, transcription-dependent R-loops persist in mitosis especially at loci such as long genes at common fragile sites that replicate in late S-G2 phase. By early mitosis, FANCD2 (depicted as D2), which is able to bind R-loop structures in complex with FANCI^50^, is recruited to these loci, visible as gaps on mitotic chromosomes. FANCD2 promotes MUS81 and XPF-dependent cleavages and rescue of un-replicated DNA structures by mitotic DNA synthesis (MiDAS), which allows sister-chromatid disjunction and faithful chromosome segregation. In absence of FANC pathway, these structures remain unresolved and un-replicated DNA impairs chromosome segregation causing mitotic failure and cell death.

We have also shown that BIR-like intermediates and MiDAS are fully dependent on MUS81 activity. Since SLX4 is a scaffold for both XPF and MUS81, and is also involved in rescue of under-replicated DNA in mitosis^26,69^, it may coordinate both activities in conjunction with FANCD2. Thus, we propose that unresolved DNA structures in SETX-depleted mitotic cells result from replication forks stalled by transcription complexes or R-loops. This type of structures^58^ would require XPF on the one side and MUS81 on the other, to re-establish replication forks and resume DNA synthesis, which limits chromosome under-replication and non-disjunction. FANCD2 is key to allow these DNA transitions to occur since it is involved in both limiting fragile chromatin and promoting breaks induction and EdU incorporation which, as we have shown, also limits chromosome segregation defects (Fig. 6). Our work adds mechanistic insights and lends support to a recent report showing that the FA pathway is an intrinsically BIR-like pathway to deal with replication stress^70^. We show here that persistent transcription-associated RS is counteracted by FANCD2 that promotes SSE cleavage and BIR-mediated rescue of under-replicated DNA during mitosis, a mechanism essential to enable faithful chromosome segregation. It will be important in the future to understand how FANCD2 functionally interacts with SLX4, and with the other members of the FA pathway, to promote SSE-mediated resolution of these structures.

Finally, the synthetic proliferation defect and the difference in the impact of SETX depletion in cancerous versus the non-cancerous cell lines hint towards therapeutic strategies targeting endogenous RS in tumors, especially in specific genetic backgrounds^71–73^ or contexts characterized by increased TRCs, such as following oncogene activation, metabolic stress or estrogen-stimulated transcription^74–76^. For example, even if germline mutations in *SETX* associated with neurodegenerative disorders do not reportedly increase cancer risk, *SETX* somatic mutations and CNVs are commonly found in tumors, particularly in gynecological and colon cancers (data from The Cancer Genome Atlas (TGCA) Program)^77^. In addition, based on our data, FA-deficient tumors should be highly dependent on SETX-mediated TRC resolution to prevent excessive mitotic abnormalities and cell death. Though further work will be necessary, we anticipate that the synthetic lethal interaction we uncovered between SETX and FA proteins may direct new strategies for cancer treatment, with similar therapeutic applications as the successful use of PARP inhibitors in BRCA-mutant tumors.

## Methods

### Cell line, culture conditions and treatments

Human colon cancer cell line, HCT116 (American Type Culture Collection), was maintained in McCoy’s 5A medium (Thermo Fisher Scientific), Human cervical cancer cell line, HeLa (American Type Culture Collection), was maintained in DMEM medium (Thermo Fisher Scientific) and the hTERT immortalized retinal pigmented epithelium cell line, hTERT RPE-1 (American Type Culture Collection) and MCF7 (ATCC HTB-22) were maintained in DMEM:F12 medium (Thermo Fisher Scientific). MCF10-2A (ATCC CRL-10781) were cultured in DMEM:F12 medium supplemented with 500 ng/mL hydrocortisone, 20 ng/mL hEGF, 0,01 mg/mL insulin, 100 ng/ml cholera toxin, 5% horse serum and 1x PSF (penicillin-streptomycin-fungizone). Head and neck squamous cell carcinoma (HNSCC) FaDu FANCA+/+ parental cell line, FANCA−/− (FA-KO) and FA-KO + FANCA transgene (FA-corr) clonal sublines obtained from The Fanconi Anemia Cancer Cell Line Resource and validated for FANCA status were grown in Eagle’s Minimum Essential Medium.

Except for MCF10-2A, all media were supplemented with 10% fetal bovine serum (FBS), 1 mM sodium pyruvate, 100 U/mL penicillin and 100 μg/mL streptomycin. All cells were cultured at 37 °C in a humidified atmosphere under 5% CO_2_.

Triptolide was used at a final concentration of 1 μM for final 8 h of culture, whereas DRB was used at a final concentration of 20 μM for final 16 h. Drugs were washed out before proceeding with the experiment.

### siRNA and plasmid transfection

siRNA duplex oligonucleotides targeting LacZ, FANCD2, SETX, XPF, MUS81 were transfected with siRNAs (sequences below) at a final concentration of 50 nM using Interferin (Polyplus) according to manufacturer’s instructions. Unless otherwise specified, siSETX#1 was used for SETX-depletion. After 72 h of incubation with siRNA, protein expression was assessed by immunoblotting. Plasmid transfections were carried out by using the Jet-PEI transfection reagent (Polyplus).

### siRNA sequences

siLacZ: CGU-CGA-CGG-AAU-ACU-UCG-A

siFANCD2: GGA-GAU-UGA-UGG-UCU-ACU-A

siSETX#1: GCC-AGA-UCG-UAU-ACA-AUU-A

siSETX#2: CCA-CCT-GGC-GTA-CAC-CAA-C

siMUS81: SMARTpool (M-016143, Dharmacon) GGG-AGC-ACC-UGA-AUC-CUA-A, CAG-GAG-CCA-UCA-AGA-AUA-A, GGG-UAU-ACC-UGG-UGG-AAG-A, CAG-CCC-UGG-UGG-AUC-GAU-A

siXPF: GUA-GGA-UAC-UUG-UGG-UUG-A

### Immunoblotting

Cells were collected by trypsinization, washed with cold PBS, re-suspended in SDS–polyacrylamide gel electrophoresis loading buffer (0.05 M Tris–HCl pH 6.8, 2% SDS, 10% glycerol, 0.01% bromophenol blue, 50 mM DTT) and passed through a needle and heated at 95 °C for 10 min. Samples were separated on SDS-PAGE denaturing gels (Bio-Rad) and transferred to nitrocellulose membranes (Bio-Rad). Membranes were blocked with PBS-Milk (5%) for 1 h before being incubated in the indicated primary antibody and the corresponding secondary antibody. Signals were visualized with WesternBright ECL (Advansta) on a digital imaging system (GeneGnome, Syngene) or using Amersham Imager 600 (GE).

Antibodies used: Vinculin (Abcam ab18058), FANCD2 (Abcam ab108928), SETX (Novus Biologicals NB100-57542), MUS81 (Abcam ab14387), XPF (Invitrogen MA5-12054).

### Clonogenic assays

Cells were plated at densities between 500 and 750 cells per well in 6-well plates. Colonies were fixed with methanol for 10 min and stained with 1% Crystal violet (Sigma) in ethanol. 6-well plates were then scanned and colonies counted. Clonogenic survival was expressed relative to control cells.

### Flow cytometry

Cells were trypsinized, washed with PBS, and centrifuged 10 min at 800 rpm. Cells were then fixed in ice-cold 70% ethanol and incubated at 4 °C overnight. Cell were centrifuged 10 min at 1000 rpm and the pellet resuspended in PI/RNase staining buffer (BD Bioscience, # 550825) and analyzed by flow cytometry (BD Accuri C6) as previously reported^78^.

### Immunofluorescence

Cells grown on glass coverslips were fixed in 4% paraformaldehyde for 15 min before permeabilization with 0.5% Triton-X 100 for 10 min at room temperature. Coverslips were then blocked in 3% BSA in PBS containing 0.05% Tween-20, cells were stained overnight at 4 °C with the primary antibody against FANCD2 (Abcam) followed by a secondary anti-rabbit Alexa Fluor 488 or 594 (Invitrogen) antibody for 1 h at room temperature. Dried coverslips were mounted on microscope slides using the VECTASHIELD Antifade Mounting Medium with DAPI (Vector laboratories) and examined at x63 magnification using an epifluorescence microscope (Zeiss Axio Observer Z1) equipped with an ORCA-ER camera (Hamamatsu). Image processing and analysis were performed using ImageJ software.

### Metaphase spread preparation

Cells were treated with 100 ng/mL colcemid (Roche) for the last 3 h. Cells were then trypsinized, collected into tubes and subjected to hypotonic shock solution (0.075 M KCl: FBS: H_2_O at a ratio of 1:1:5) for 15 min at 37 °C followed by fixative solution (ethanol:acetic acid at a ratio of 3:1) overnight at −20 °C. The next day cells were centrifuged at 1200 rpm for 5 min and resuspended in fixative solution, dropped onto slides and air dried until the next day. Slides were then stained with VECTASHIELD Antifade Mounting Medium with DAPI (Vector laboratories) and metaphase spreads examined x63 magnification using an epifluorescence microscope (Zeiss Axio Observer Z1) equipped with an ORCA-ER camera (Hamamatsu).

### Metaphase spread preparation for FISH experiments

HCT116 cells were treated with 100 ng/mL colcemid (Roche) for 3 h. After trypsinization, cells were centrifuged into 15 ml tubes for 5 min prior adding of hypotonic shock solution (0.75 M KCl:RNase free H_2_O at a ratio of 1:10) for 15 min at 37 °C. Swollen cells were centrifuged and resuspended twice with cold fixative solution (methanol:acetic acid at a ratio of 3:1) and then stored overnight at −20 °C. The next day cells were centrifuged at 1200 rpm for 5 min and resuspended in fixative solution, dropped onto slides (washed in absolute Ethanol) and air dried until the next day.

For the recognition of fragile chromatin, metaphase spreads were stained before with Giemsa (Carlo Erba). After the discoloration with fixative solution, the chromosomes were stained with a solution of DAPI (4′,6′-diamidino-2-phenylindole hydrochloride, 1 μg/mL; Sigma) and VECTASHIELD Antifade Mounting Medium (Vector Laboratories) (DAPI: VECTASHIELD Antifade Mounting Medium at a ratio of 1:300) and observed with a x100 magnification at epifluorescence microscope (Nikon, Minato, Tokyo, Japan) equipped with an HBO 100-W mercury lamp a Qimaging camera (QICAM Mono Fast 1394 Cooled).

Metaphase spreads with Fluorescence In Situ Hybridization (FISH) were observed with a ×63 magnification at epifluorescence microscope (Zeiss Axio Observer Z1) with an ORCA-ER camera (Hamamatsu). The slides were stored in the dark at 4 °C.

Greyscale images for fluorophore and DAPI signals were acquired separately, pseudo-colored, and merged using the Photoshop software.

### BAC extraction and labeling by nick translation and random priming

The bacterial artificial chromosomes (BAC) was selected from GenBank (https://www.ncbi.nlm.nih.gov/genbank/) for FRA3B fragile region on chromosome 3 (RP11-48E21 AC093556, chr3: 60,541,107-60,572,598).

Bacterial cells were grown in 10 mL of Luria-Bertani (LB) medium with the addition of 20 μg/mL chloramphenicol. Alkaline lysis protocol was performed for BACs extraction.

The extracted BACs were labeled with bio-16-dUTP (biotin-16-deoxy-Uridine Triphosphate) and/or dig-16-dUTP (digoxigenin-16-deoxy-Uridine Triphosphate) by Nick Translation; the extracted BACs were also labeled with bio-14-dCTP (biotin-14-deoxy-Cytidine Triphosphate) by Random Priming kit (BioPrime™ DNA Labeling System Invitrogen 18094011). The labeled probes were used for fluorescent in situ hybridization (FISH) experiments on metaphase spreads.

### Combined DNA-RNA FISH

The slides were dehydrated with 70%, 90%, and 100% ethanol for 5 min each wash. Slides were aged at 65 °C for 60 min. Meanwhile, BAC RP11-48E21 AC093556 (FRA3B) was denatured at 80 °C for 8 min and subsequently incubated for 15 min at 37 °C.

The sample was denatured at 70 °C for 2 min with 70% deionized formamide (Sigma) 2× SSC (Saline Sodium Citrate), after the denaturation, the slides were washed with cold 70% ethanol for 5 min and dehydrated again with 90 and 100% ethanol solution for 5 min each wash and air dried. On each slide 200 ng of denatured probe were added and were incubated overnight at 37 °C.

After three 2×SSC post-hybridization washes for 5 min each at 42 °C, the slides were incubated for 30 min with antibody anti-digoxigenin-rhodamine (1:300, Roche) or FITC-avidin (1:20). Three washes in 2× SSC 0.1% Tween20 were performed, and the slides were counterstained with VECTASHIELD Antifade Mounting Medium with DAPI (Vector laboratories). All the used solutions were prepared in RNase-free H_2_Odd, adding 0.1% DEPC (Diethyl Pyrocarbonate, Sigma).

### RNase A and RNase H treatments

After the visualization of 50 metaphases spreads and 50 nuclei with the combined DNA-RNA FISH for each biological replicate, the slides were delicately washed two times in PBS 1X at room temperature and then treated with RNase A solution (2XSSC, 0,1 mg/ml of RNase A enzyme, Thermo Fisher Scientific) for 1 h at 37 °C. Three washes in 70%, 90%, and 100% ethanol were performed and then the slides were stained with VECTASHIELD Antifade Mounting Medium with DAPI (Vector laboratories),

For RNAse H treatment, after counting FISH spots the slides were washed two times in PBS 1X at room temperature and treated with RNase H solution (PBS 1X, 10% Reaction Buffer 10X and 0.05 U/µL of RNase H enzyme, Thermo Fisher Scientific) for 1 h at 37 °C. As before, three washes in 70%, 90%, and 100% ethanol were performed and the slides were stained with VECTASHIELD Antifade Mounting Medium with DAPI (Vector laboratories). To further verify the effect of RNase H on FISH signals, RNase H solution was also used for 1 h at 37 °C to treat the slides before the three washes in 70%, 90%, and 100% ethanol and the combined DNA-RNA FISH protocol.

### EdU incorporation

To label replicating DNA in mitosis, asynchronous cells seeded on glass coverslips were incubated with 10 μM EdU for the last 1 h after siRNA transfection. Coverslips were then fixed in 4% paraformaldehyde for 15 min and incorporated EdU was detected using the Click-iT EdU Alexa Fluor 488 imaging Kit (Molecular Probes) according to manufacturer’s instructions. Dried coverslips were mounted on microscope slides using the VECTASHIELD Antifade Mounting Medium with DAPI (Vector laboratories) and mitotic cells examined ×63 magnification using an epifluorescence microscope (Zeiss Axio Observer Z1) equipped with an ORCA-ER camera (Hamamatsu).

### Statistics and reproducibility

Quantitative data are presented as the means ± SEM of at least three independent experiments for all figures. Unless otherwise stated, significance was tested using a two-tailed Student’s *t* test. Statistical tests were performed using Prism (GraphPad software). *P* values are indicated as *p ≤ 0.05, **p < 0.01, ***p < 0.001, and ****p < 0.0001, with ns indicating not significant (*p* > 0.05). Individual p values are indicated in the graphs above each corresponding condition and refer to significant difference relative to control or to the condition indicated by the black line.

### Reporting summary

Further information on research design is available in the Nature Portfolio Reporting Summary linked to this article.

## Supplementary information

Supplementary Information

Description of Additional Supplementary Files

Supplementary Data 1

Reporting Summary

## Data Availability

Unprocessed blot images are shown in Supplementary Fig. 6. Source data underlying the graphs are available in Supplementary Data 1. All other relevant data are available from the corresponding author on request.
