# Supplementary material for: FANCD2 promotes mitotic rescue from transcription-mediated replication stress in SETX-deficient cancer cells
Source: Commun Biol. 2022 Dec 21;5:1395. doi: 10.1038/s42003-022-04360-2 (PMC9772326; doi:10.1038/s42003-022-04360-2)

Supplementary Information

Supplementary Figure 1: Western blot evaluation of SETX siRNA efficiency and phenotypic characterization of SETX-deficient cells.

a) Western blot detection of FANCD2 and SETX protein levels from whole cell lysates of control (LacZ), SETX#1, and SETX#2 siRNA-transfected cells. Vinculin was used as loading control.
b, c) Quantification of the number of chromosome aberrations (gaps or under-condensed regions) in control or SETX-depleted HeLa and RPE1 cells, respectively.
d, e) Western blots showing efficiency of siRNA-mediated depletion of SETX in HeLa and RPE1 cells, respectively.
f) Cell cycle analysis showing the proportion of cells in G1, S, and G2-M phase in control (siLacZ) and SETX-depleted condition. Cells in each cell cycle phase were quantitated by flow cytometry based on DNA content following staining with propidium iodide.
g, h) Quantification of FANCD2 foci on mitotic chromosomes in HeLa and RPE1 cells, respectively.

Supplementary Figure 2: Evaluation of the effects of transcription inhibitors, RNase H1 and APH on FANCD2 protein levels and foci formation, and on FHIT foci at FRA3B in control and SETX depleted conditions (related to Figure 2).

a) Western blot evaluation of SETX and FANCD2 protein levels in control (LacZ) and SETX siRNA-transfected cells after treatment with 1 µM Triptolide (Trip) for 8 h, or 20 µM DRB for 16 h. Vinculin was used as loading control.
b) Western blot detection of GFP and RNase H1 expression levels in LacZ or SETX RNAi cells after transfection of a control (EV-GFP) or RNase H1-GFP expression vector. Vinculin was used as loading control.
c) Quantification of FANCD2-positive (>5 FANCD2 foci) cells in control (LacZ) or SETX siRNA-transfected cells following 24 h treatment with 0.3 µM APH.
d) Quantification of FHIT/FRA3B FISH signals in metaphase in control (LacZ) and SETX siRNA-transfected cells, detected on slides pre-treated with mock or RNase H solution prior to the combined RNA/DNA FISH protocol or prepared from cells

transfected with an empty or an RNase H1 over-expressing vector. 50 metaphases were counted for each condition.

Supplementary Figure 3: Western blot evaluation of SETX and FANCD2 siRNA efficiency and frequency of metaphases presenting fragile chromatin.

a) Western blot detection of FANCD2 and SETX protein levels from whole cell lysates from control (LacZ), FANCD2, SETX, and FANCD2 and SETX siRNA-transfected cells. Vinculin was used as loading control.
b) Quantification of the number of metaphases presenting the fragile chromatin phenotype in metaphase spreads prepared from control, SETX, FANCD2, and SETX and FANCD2 siRNA-transfected cells. Data are the results of duplicate experiments. A total of 43 (siLacZ), 55 (siFANCD2), 59 (siSETX), and 23 (siSETX+siFANCD2) metaphases were counted.

Supplementary Figure 4: Western blot evaluation of SETX, MUS81 and XPF siRNA efficiency.
a) Western blot detection of SETX and XPF protein levels from whole cell lysates from control, SETX, XPF, and SETX and XPF siRNA-transfected cells. Vinculin was used as loading control.
b) Western blot detection of SETX and MUS81 protein levels from whole cell lysates from control, SETX, MUS81, and SETX and MUS81 siRNA-transfected cells. Vinculin was used as loading control.

Supplementary Figure 5: Evaluation of the effect of SETX and FANCD2 single or double depletion on chromosome segregation in MCF7 cells.
Quantification of anaphases presenting bulky DNA bridges and lagging chromosomes in control, SETX, FANCD2, and SETX and FANCD2 siRNA-transfected MCF7 cells.

Supplementary Figure 6: Unprocessed blots images related to Figure 4h; Supplementary Figure 1a, d, e; Supplementary Figure 2a, b; Supplementary Figure 3; Supplementary Figure 4a, b.

Supplementary Figure 1

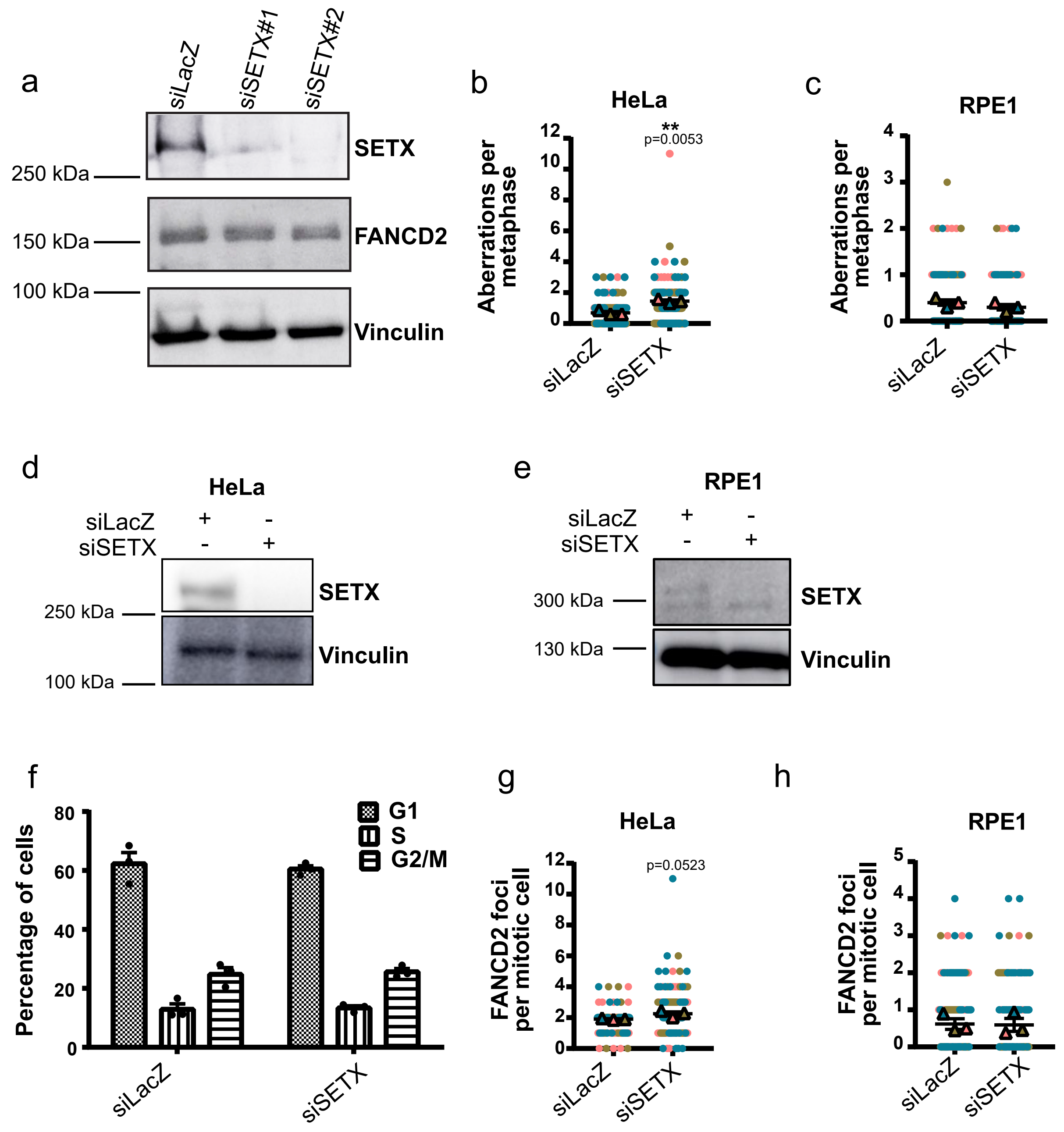

Supplementary Figure 2

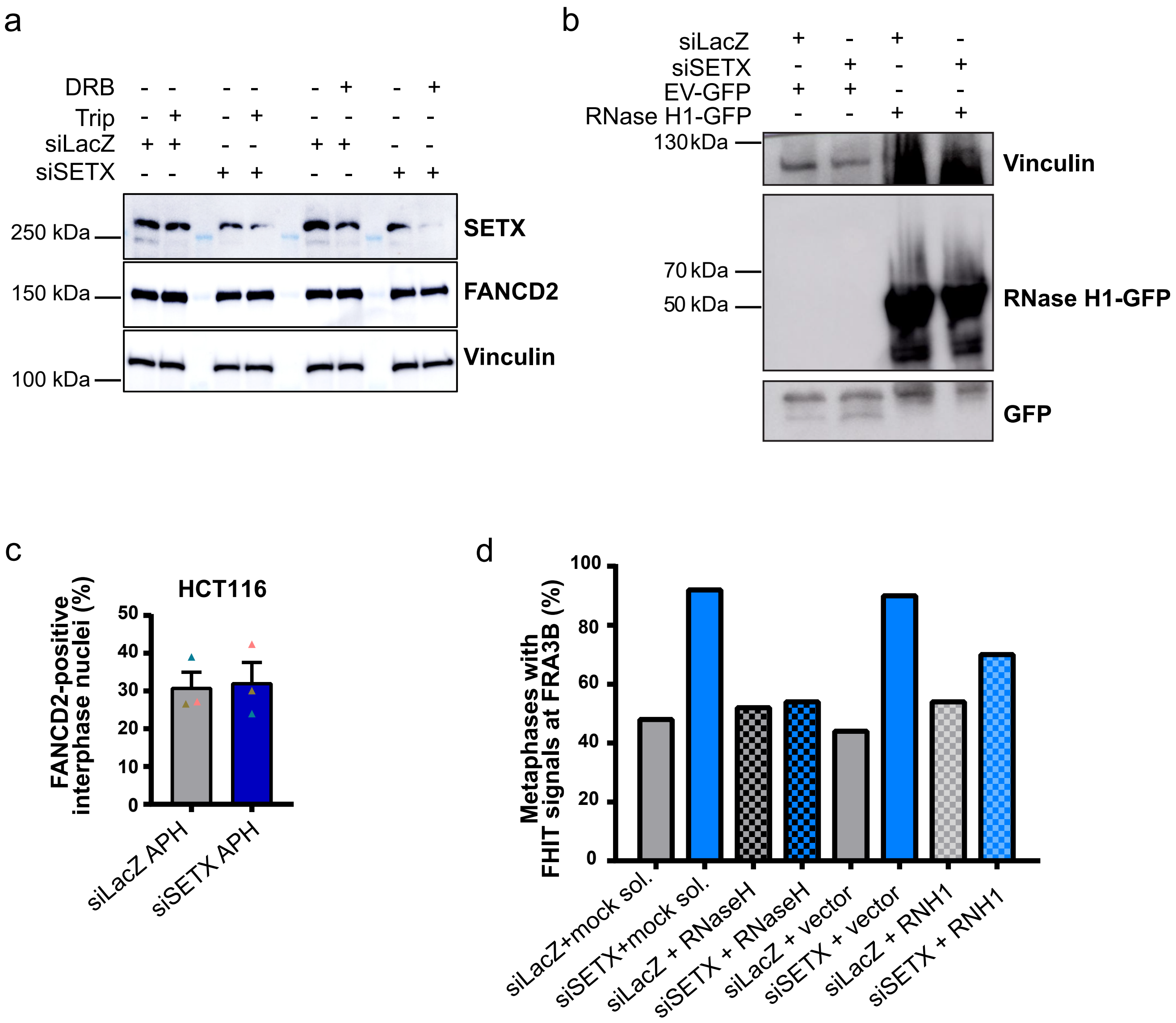

Supplementary Figure 3

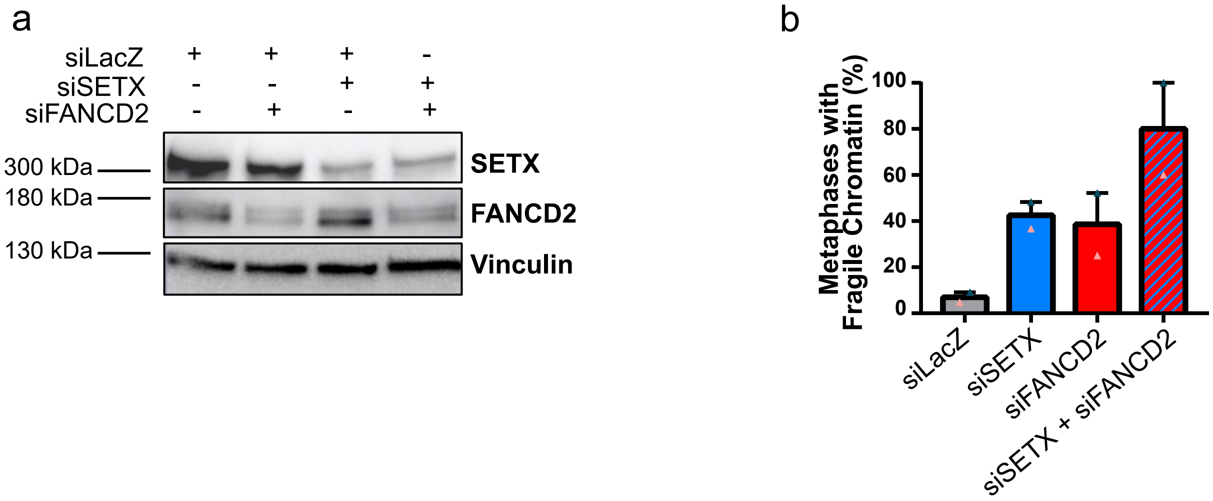

Supplementary Figure 4

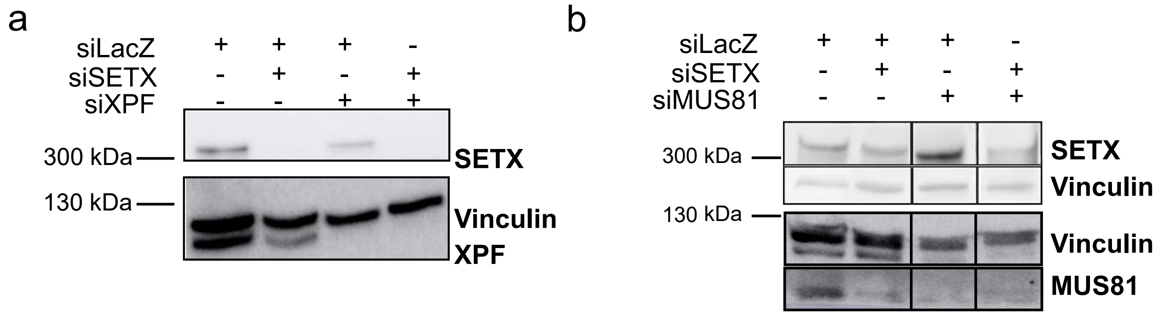

Supplementary Figure 5

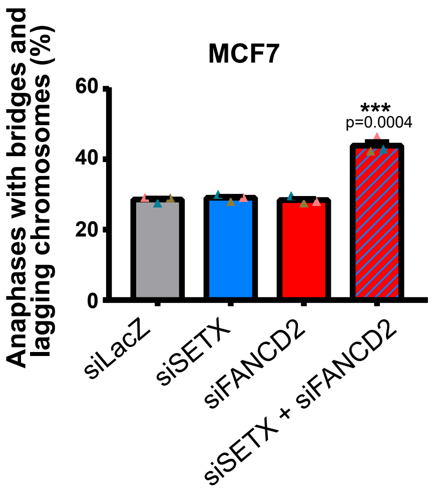

Supplementary Figure 6: unprocessed blots

related to Figure 4h

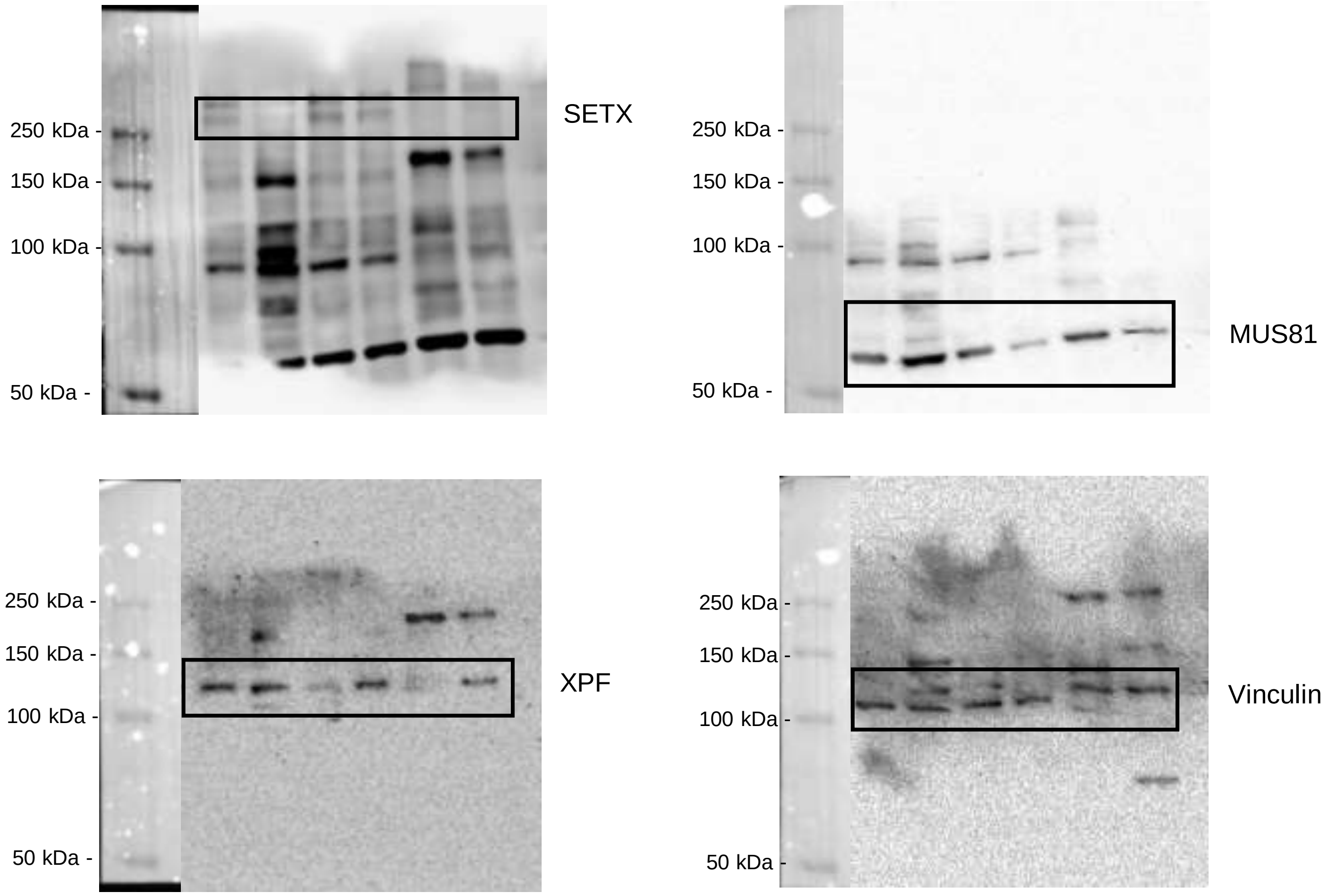

related to Supplementary Figure 1a

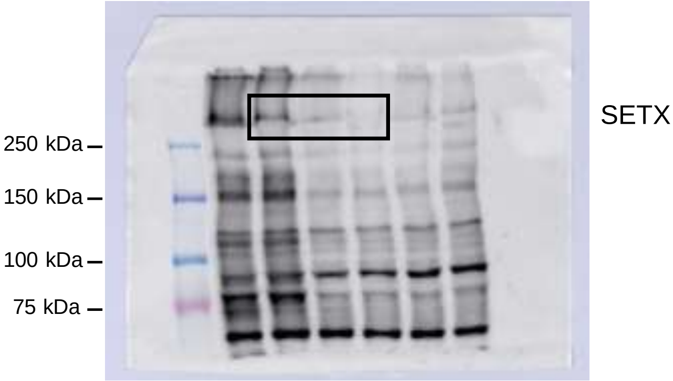

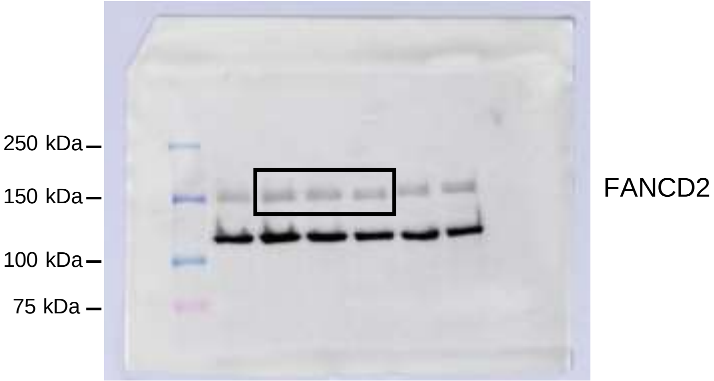

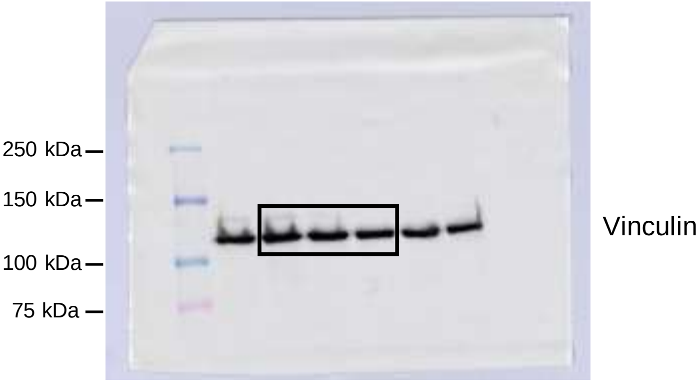

related to Supplementary Figure 1d, e

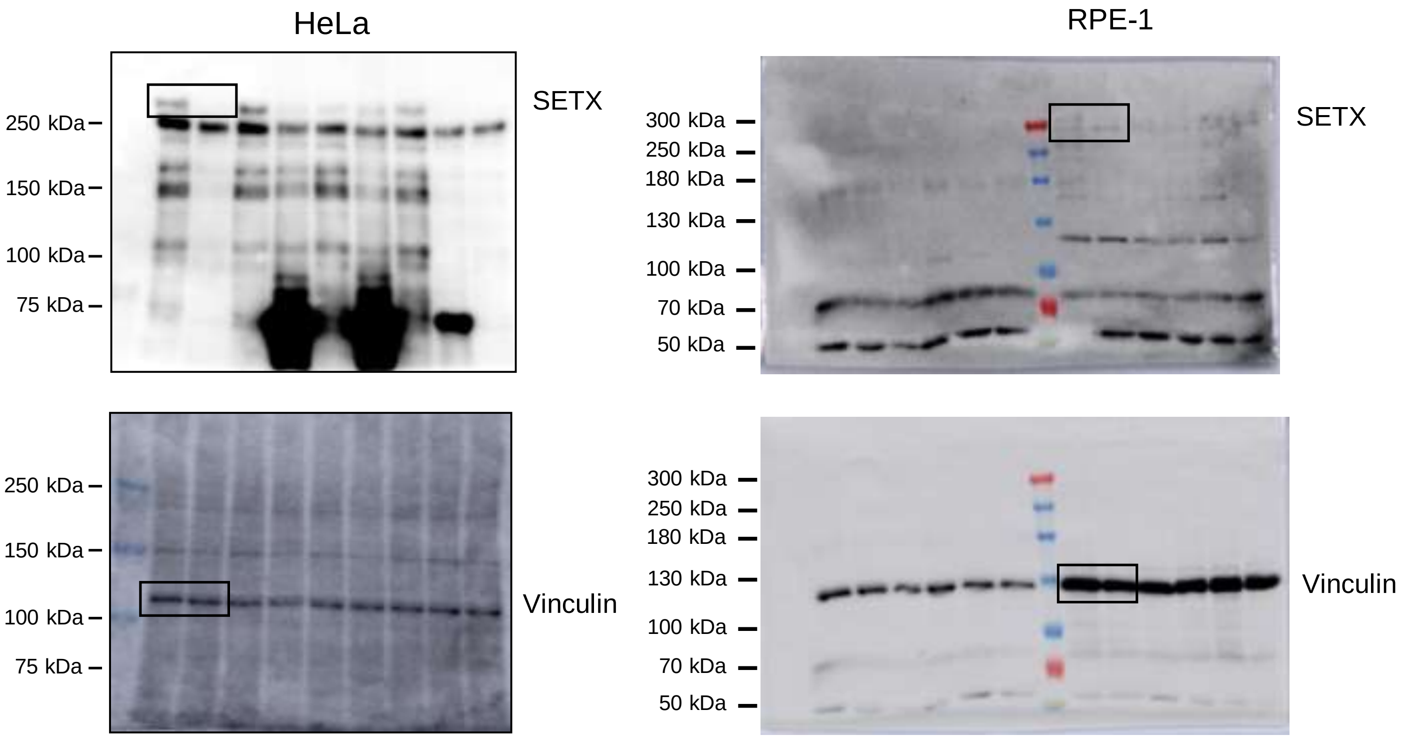

related to Supplementary Figure 2a

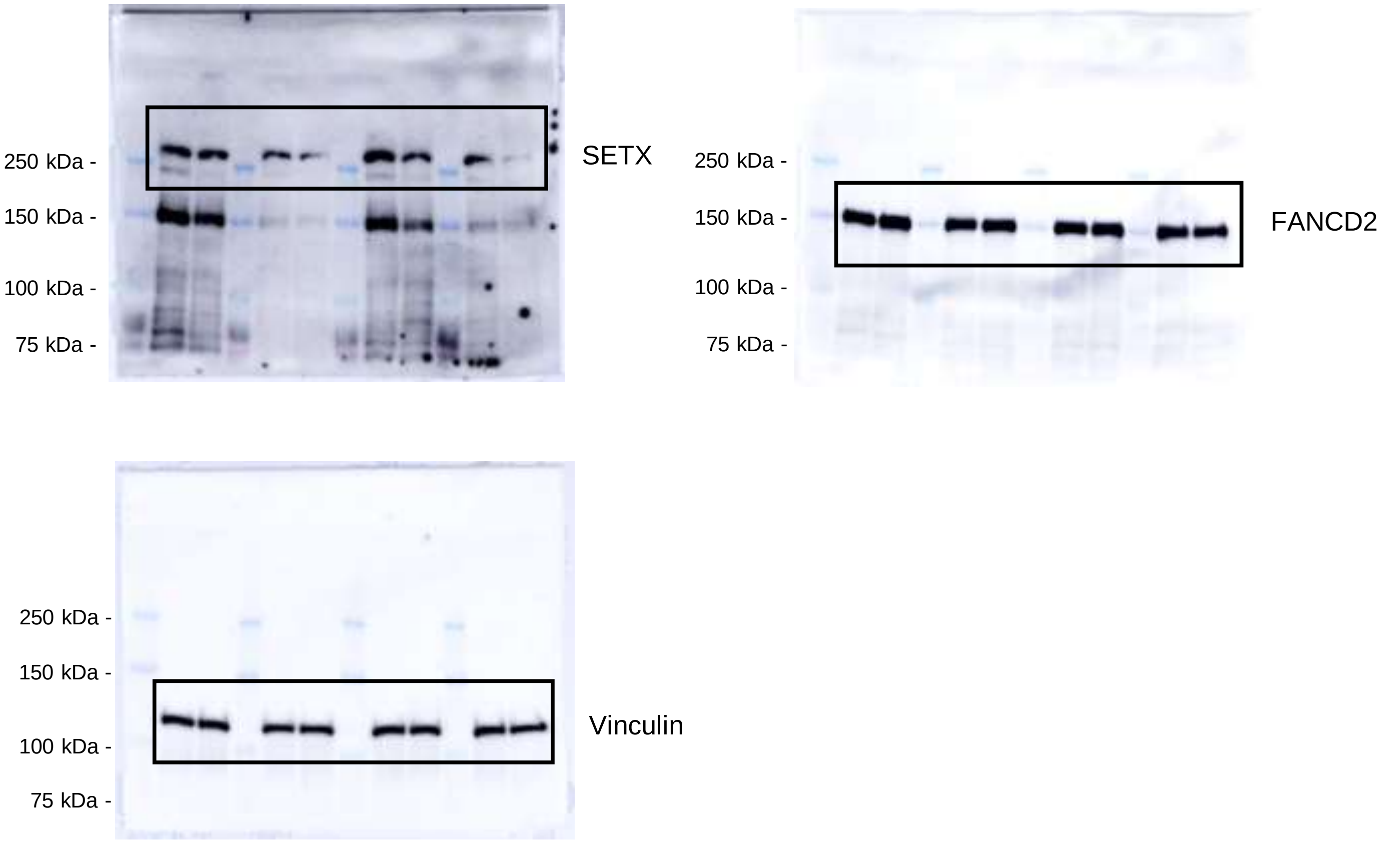

related to Supplementary Figure 2b

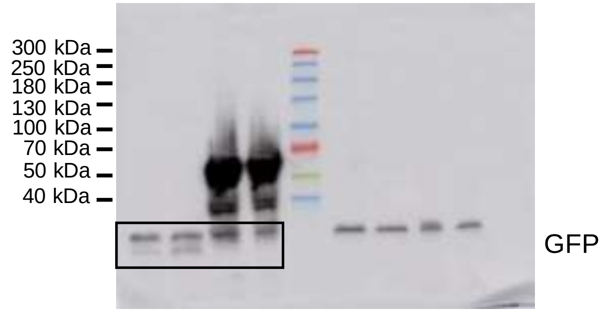

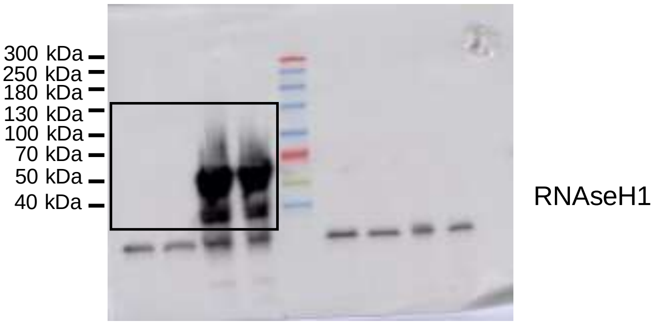

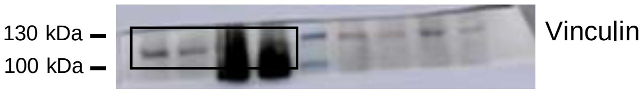

related to Supplementary Figure 3

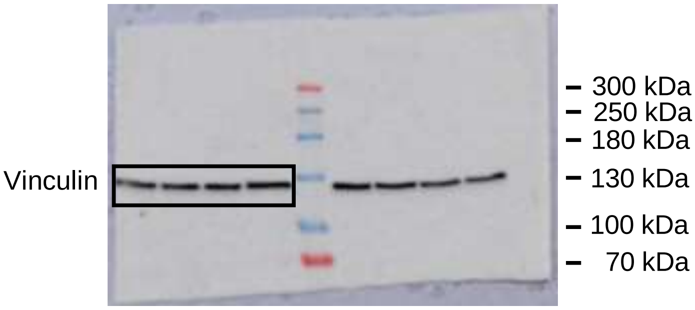

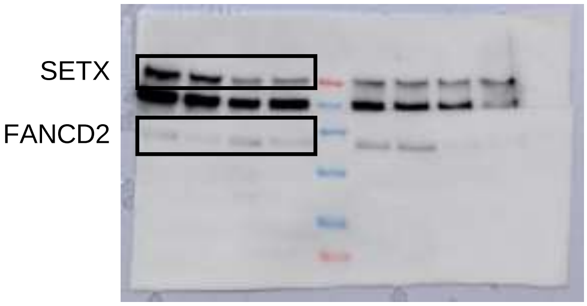

related to Supplementary Figure 4a

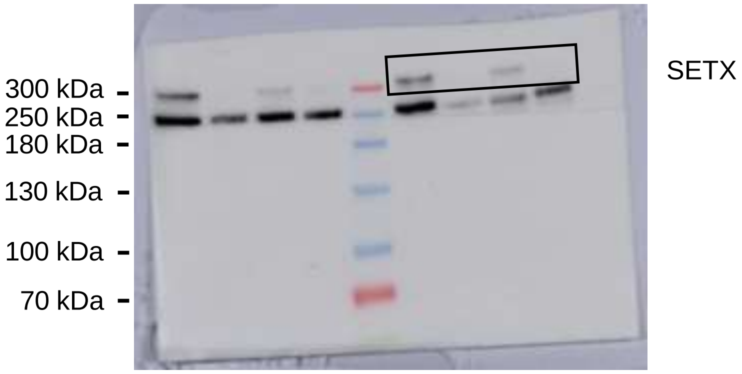

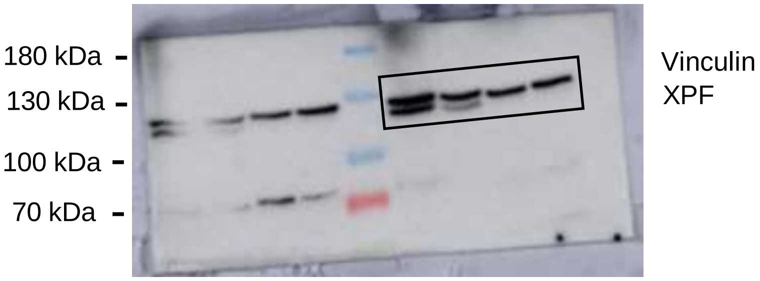

related to Supplementary Figure 4b

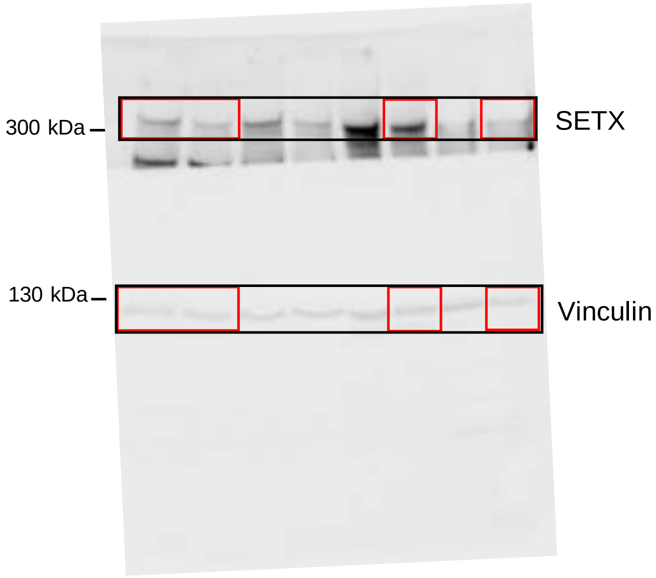

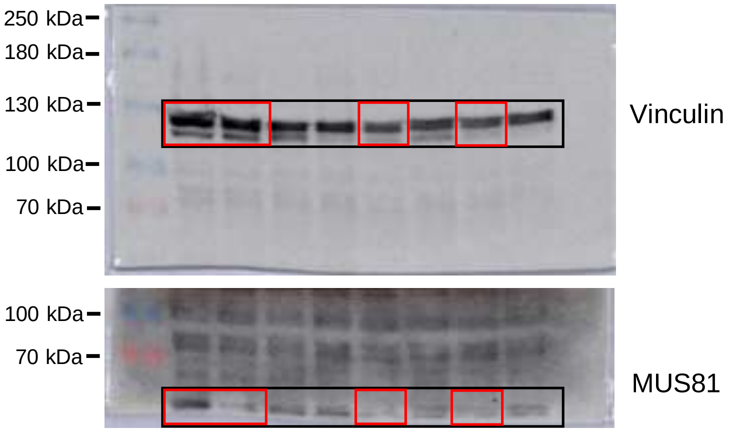

Supplement: Supplementary file 1 — Supplementary Information [file 42003_2022_4360_MOESM1_ESM.pdf]
