# Supplementary material for: FANCD2 promotes mitotic rescue from transcription-mediated replication stress in SETX-deficient cancer cells
Source: Commun Biol. 2022 Dec 21;5:1395. doi: 10.1038/s42003-022-04360-2 (PMC9772326; doi:10.1038/s42003-022-04360-2)
Supplement: Supplementary file 4 — Reporting Summary [file 42003_2022_4360_MOESM4_ESM.pdf]

nature research

Corresponding author(s): Valeria Naim

Last updated by author(s): 30 Nov 2022

Reporting Summary

Nature Research wishes to improve the reproducibility of the work that we publish. This form provides structure for consistency and transparency in reporting. For further information on Nature Research policies, see Authors & Referees and the Editorial Policy Checklist.

Statistics

For all statistical analyses, confirm that the following items are present in the figure legend, table legend, main text, or Methods section.

n/a	Confirmed	
☐	☒	The exact sample size (n) for each experimental group/condition, given as a discrete number and unit of measurement
☐	☒	A statement on whether measurements were taken from distinct samples or whether the same sample was measured repeatedly
☐	☒	The statistical test(s) used AND whether they are one- or two-sided<br>*Only common tests should be described solely by name; describe more complex techniques in the Methods section.*
☒	☐	A description of all covariates tested
☒	☐	A description of any assumptions or corrections, such as tests of normality and adjustment for multiple comparisons
☐	☒	A full description of the statistical parameters including central tendency (e.g. means) or other basic estimates (e.g. regression coefficient) AND variation (e.g. standard deviation) or associated estimates of uncertainty (e.g. confidence intervals)
☐	☒	For null hypothesis testing, the test statistic (e.g. F, t, r) with confidence intervals, effect sizes, degrees of freedom and P value noted<br>*Give P values as exact values whenever suitable.*
☒	☐	For Bayesian analysis, information on the choice of priors and Markov chain Monte Carlo settings
☒	☐	For hierarchical and complex designs, identification of the appropriate level for tests and full reporting of outcomes
☒	☐	Estimates of effect sizes (e.g. Cohen's d, Pearson's r), indicating how they were calculated

Our web collection on statistics for biologists contains articles on many of the points above.

Software and code

Policy information about availability of computer code

Data collection	Not applicable
Data analysis	Not applicable

For manuscripts utilizing custom algorithms or software that are central to the research but not yet described in published literature, software must be made available to editors/reviewers. We strongly encourage code deposition in a community repository (e.g. GitHub). See the Nature Research guidelines for submitting code & software for further information.

Data

Policy information about availability of data

All manuscripts must include a data availability statement. This statement should provide the following information, where applicable:

- Accession codes, unique identifiers, or web links for publicly available datasets
- A list of figures that have associated raw data
- A description of any restrictions on data availability

Source data will be available in Supplementary Data 1

Field-specific reporting

Please select the one below that is the best fit for your research. If you are not sure, read the appropriate sections before making your selection.

☒ Life sciences ☐ Behavioural & social sciences ☐ Ecological, evolutionary & environmental sciences

For a reference copy of the document with all sections, see nature.com/documents/nr-reporting-summary-flat.pdf

October 2018

Life sciences study design

All studies must disclose on these points even when the disclosure is negative.

Sample size	No statistical methods were used to determine sample size.
Data exclusions	No data was excluded from the analysis.
Replication	All data are the results of at least three independent experiments and n is indicated in the Figure legends.
Randomization	Not applicable
Blinding	Investigators were not blinded.

Reporting for specific materials, systems and methods

We require information from authors about some types of materials, experimental systems and methods used in many studies. Here, indicate whether each material, system or method listed is relevant to your study. If you are not sure if a list item applies to your research, read the appropriate section before selecting a response.

Materials & experimental systems

n/a	Involved in the study	
☐	☒	Antibodies
☐	☒	Eukaryotic cell lines
☒	☐	Palaeontology
☒	☐	Animals and other organisms
☒	☐	Human research participants
☒	☐	Clinical data

Methods

n/a	Involved in the study	
☒	☐	ChIP-seq
☐	☒	Flow cytometry
☒	☐	MRI-based neuroimaging

Antibodies

Antibodies used	All the antibodies used in the study are indicated in the Methods section.
Validation	All the antibodies were validated by the suppliers and in previous publications.

Eukaryotic cell lines

Policy information about cell lines

Cell line source(s)	All cell lines were purchased from ATCC, except FaDu cells that were obtained by Fanconi Anemia Cancer Cell Line Resource.
Authentication	Cell lines were authenticated by ATCC. FaDu cells were validated by Fanconi Anemia Cancer Cell Line Resource.
Mycoplasma contamination	All cell lines were tested negative for Mycoplasma contamination.
Commonly misidentified lines (See ICLAC register)	No misidentified lines were used.

Flow Cytometry

Plots

Confirm that:

☐ The axis labels state the marker and fluorochrome used (e.g. CD4-FITC).

☐ The axis scales are clearly visible. Include numbers along axes only for bottom left plot of group (a 'group' is an analysis of identical markers).

☐ All plots are contour plots with outliers or pseudocolor plots.

☒ A numerical value for number of cells or percentage (with statistics) is provided.

Methodology

Sample preparation	Cells were trypsinized, washed with PBS, and centrifuged 10 min at 800 rpm. Cells were then fixed in ice-cold 70% ethanol and

Sample preparation	incubated at 4°C overnight. Fixed cells were then washed in PBS and resuspended in PBS containing propidium iodide (30 μg/mL) and RNAse (100 μg/mL) and analyzed by flow cytometry.
Instrument	BD Accuri C6
Software	BD Accuri C6 software files were exported in FCS 3.1 format and analyzed using the FlowJo software.
Cell population abundance	For each sample, 20,000 events were collected and acquired by FACS.
Gating strategy	The discrimination of cell doublets from singlets was made by plotting the DNA dye (PI) channel Width versus Area in a dot plot graph. The gating was done by distinguishing G1, S and G2/M phase on the base of DNA content (PI staining). PI intensity was detected by FL3-A filters.

☐ Tick this box to confirm that a figure exemplifying the gating strategy is provided in the Supplementary Information.
